# Non-invasive testing in metabolic dysfunction-associated steatotic liver disease

**DOI:** 10.3389/fmed.2024.1499013

**Published:** 2024-11-13

**Authors:** Sanad Dawod, Kimberly Brown

**Affiliations:** Henry Ford Hospital, Detroit, MI, United States

**Keywords:** MASLD, non-invasive testing, NAFLD, MASH, liver fibrosis

## Abstract

Metabolic dysfunction-associated steatotic liver disease (MASLD), previously referred to as non-alcoholic fatty liver disease (NAFLD), is a leading cause of chronic liver disease, affecting up to 30% of the global population. MASLD is strongly associated with metabolic risk factors such as obesity and type 2 diabetes, and can progress to advanced stages including cirrhosis and hepatocellular carcinoma. Early diagnosis and accurate staging of fibrosis are critical in managing the disease and preventing complications. While liver biopsy has long been considered the gold standard for assessing fibrosis, it is invasive and carries associated risks. In response, non-invasive tests (NITs) have emerged as essential alternatives for the diagnosis and monitoring of MASLD. Key methods include blood-based biomarkers such as the Fibrosis-4 (FIB-4) score, NAFLD Fibrosis Score (NFS), and Enhanced Liver Fibrosis (ELF) test, as well as imaging modalities like vibration-controlled transient elastography (VCTE) and magnetic resonance elastography (MRE). These tests provide safer, more accessible methods for identifying liver fibrosis and guiding clinical management. They are integral in assessing disease severity, guiding treatment decisions, and monitoring disease progression, particularly in light of emerging therapies. NITs have become increasingly recommended by clinical guidelines as they reduce the need for invasive procedures like liver biopsy, improving patient care and outcomes. In conclusion, non-invasive testing plays a crucial role in the effective management of MASLD, offering reliable alternatives for diagnosis and monitoring while minimizing risks associated with traditional invasive methods.

## Introduction

Metabolic dysfunction-associated steatotic liver disease (MASLD), previously referred to as non-alcoholic fatty liver disease (NAFLD), is one of the most common causes of chronic liver disease worldwide (1). Affecting up to 30% of the global adult population, MASLD is often associated with metabolic risk factors such as obesity, T2DM, and insulin resistance, which collectively contribute to its rising incidence (2, 3). MASLD encompasses a spectrum of conditions ranging from simple hepatic steatosis to metabolic dysfunction-associated steatohepatitis (MASH), which can progress to fibrosis, cirrhosis, and hepatocellular carcinoma (1).

The need for early detection and accurate staging of fibrosis is critical in preventing the progression of MASLD and its complications, including liver failure and HCC (4, 5). Liver biopsy is considered the gold standard for diagnosing and staging MASLD, particularly for identifying MASH and assessing fibrosis. Its utility lies in providing detailed histological information, including the degree of steatosis, inflammation, hepatocellular ballooning, and fibrosis, which are critical for determining disease severity and guiding treatment decisions. However, liver biopsy is an invasive procedure with several downsides. It carries risks such as pain, bleeding, and infection, with significant bleeding occurring in approximately 0.5% of cases and a mortality rate of about 0.01%. Additionally, liver biopsy is subject to sampling error and interobserver variability, affecting diagnostic accuracy. Despite these limitations, it remains an indispensable tool in clinical practice and research, particularly when non-invasive methods are inconclusive or insufficient (6–10).

Consequently, non-invasive tests (NITs) have gained prominence, providing reliable, repeatable, and less risky alternatives to liver biopsy. Major scientific societies, including the American Association for the Study of Liver Diseases (AASLD) and the European Association for the Study of the Liver (EASL), now recommend using non-invasive methods for diagnosing and monitoring MASLD (11–13).

Non-invasive tests play a crucial role in the management of MASLD by providing a safer, more patient-friendly alternative to liver biopsy. NITs are essential for risk stratification. They help identify patients at low, intermediate, or high risk for advanced fibrosis, which is a critical determinant of long-term outcomes in MASLD. NITs are utilized in stepwise framework for the evaluation of MASLD. They are increasingly utilized in guiding risk stratification, referral decisions, and further testing (14, 15). Additionally, given the chronic and progressive nature of MASLD, and with new emerging therapies, there is a need for monitoring disease progression and treatment response (16).

In this review, we aim to explore and evaluate the various non-invasive tests (NITs) available for diagnosing and managing MASLD focusing on their diagnostic accuracy, clinical utility, and potential for guiding treatment strategies.

## Blood-based non-invasive tests

### Blood-based calculated scores

Blood-based non-invasive tests (NITs) have become a cornerstone in the diagnosis and monitoring of MASLD. The most commonly used blood-based NITs include serum biomarkers such as the Fibrosis-4 (FIB-4) score, the NAFLD fibrosis score (NFS), and the Enhanced Liver Fibrosis (ELF) test, which have been extensively validated for use in clinical practice. These tests offer a cost-effective and accessible means of identifying patients at risk for advanced fibrosis and guiding clinical decisions.

#### Fibrosis-4 (FIB-4) and NAFLD fibrosis score (NFS)

The FIB-4 score is one of the most widely used blood-based biomarkers in the management of MASLD. It incorporates simple clinical parameters such as age, aspartate aminotransferase (AST), alanine aminotransferase (ALT), and platelet count to estimate the risk of liver fibrosis. FIB-4 has been validated across various populations and has shown a high negative predictive value (NPV) for ruling out advanced fibrosis (F3-F4), especially in primary care settings where liver biopsy may not be feasible (17, 18).

The AASLD and the EASL recommend FIB-4 as an initial screening tool, particularly in patients with metabolic risk factors such as obesity and T2DM (18).

While FIB-4 is highly effective in ruling out advanced fibrosis, its ability to detect early-stage fibrosis (F0-F2) is limited. Many patients with intermediate FIB-4 scores require further testing using more advanced imaging modalities such as vibration-controlled transient elastography (VCTE) or magnetic resonance elastography (MRE) (15, 18). Similarly, the NAFLD fibrosis score (NFS) is another widely used blood-based test that combines clinical parameters, including body mass index (BMI), fasting glucose levels, and liver enzymes, to estimate the likelihood of advanced fibrosis. The cost-effectiveness of these tests is well-documented. The FIB-4 and NFS are inexpensive and easy to calculate using standard laboratory tests, making them ideal for use in primary care settings (17–20).

#### SAFE score

The Steatosis-Associated Fibrosis Estimator (SAFE) score is a non-invasive tool for assessing liver fibrosis in patients with MASLD. It incorporates clinical variables like age, body mass index (BMI), diabetes status, platelet count, aspartate aminotransferase (AST), alanine aminotransferase (ALT), and globulin levels (21). The SAFE score is particularly useful in primary care for identifying low-risk patients who do not require invasive testing and distinguishing between mild (F0-F1) and significant fibrosis (≥F2) (22). It is most beneficial for patients with NAFLD, particularly those with metabolic risk factors like obesity T2DM (21, 22). While it has shown better performance than other blood-based markers like FIB-4 and the NFS, it still requires further validation in diverse populations (23).

### Blood-based biomarkers

#### Enhanced liver fibrosis (ELF) test

The Enhanced Liver Fibrosis (ELF) test is a more advanced serum biomarker that measures direct markers of fibrosis, including hyaluronic acid, procollagen III amino-terminal peptide (PIIINP), and tissue inhibitor of metalloproteinases-1 (TIMP-1). These markers reflect changes in the extracellular matrix during the fibrogenesis process, providing a more direct assessment of fibrosis compared to FIB-4 and the NAFLD Fibrosis Score, which rely on indirect markers of liver injury and function (24, 25). It is often employed when initial non-invasive tests like FIB-4 or NAFLD Fibrosis Score yield indeterminate results, or when there is a need for more specific fibrosis staging (12). Studies have shown that the ELF test has higher sensitivity and specificity for detecting advanced fibrosis and predicting liver-related outcomes, such as progression to cirrhosis and liver-related complications, compared to FIB-4 and the NAFLD Fibrosis Score (26, 27). The ELF test has been validated for its superior diagnostic performance in various populations, including those with MASLD and chronic hepatitis B (28, 29).

A limitation of the ELF test is its reduced specificity in low-prevalence settings, which can lead to false positives. This is particularly relevant in primary care settings where the prevalence of advanced fibrosis is lower (30, 31).

#### FibroSure

FibroSure (also known as FibroTest) is a NIT developed to assess liver fibrosis and necro-inflammatory activity, initially in chronic hepatitis B and C. It combines several serum markers, including alpha-2-macroglobulin, haptoglobin, apolipoprotein A1, gamma-glutamyl transpeptidase (GGT), and total bilirubin, into a proprietary algorithm to generate a fibrosis score. In one meta-analysis, it has been shown to have high diagnostic accuracy for detecting cirrhosis (AUC 0.92). However, it is less reliable in detecting advanced (F3-F4) and significant fibrosis (F2-F4), with an AUC of 0.77 (32, 33).

Limitations of FibroSure include its moderate sensitivity and specificity. Additionally, factors such as acute inflammation and hemolysis can affect its accuracy. Therefore, it is recommended to use FibroSure in combination with other diagnostic modalities like VCTE or ELF to enhance overall diagnostic performance and provide a more comprehensive assessment (32, 34).

### Additional blood-based tests

In addition to FIB-4, NFS, and ELF, other blood-based biomarkers such as the aspartate aminotransferase to platelet ratio index (APRI) and the Forns index are being explored for their potential utility in diagnosing and monitoring MASLD. However, these tests are less commonly used than FIB-4 and ELF due to their lower sensitivity and specificity in detecting advanced fibrosis (18, 35).

Another promising area of research involves the use of circulating microRNAs and exosomal markers as novel non-invasive biomarkers for MASLD. MicroRNAs are small, non-coding RNA molecules that regulate gene expression and are involved in various physiological and pathological processes, including liver fibrosis. Early studies suggest that certain microRNAs may serve as biomarkers for liver injury and fibrosis, potentially offering a more accurate and non-invasive way to diagnose and monitor MASLD progression. Although these markers are still in the experimental phase, they hold promise for future integration into clinical practice, particularly as more data become available from ongoing research (15, 36, 37).

## Imaging-based non-invasive tests

### Vibration-controlled transient elastography (VCTE)

FibroScan, or vibration-controlled transient elastography (VCTE), is a non-invasive imaging technique used to assess liver stiffness, which correlates with the degree of fibrosis. It is intended for use in patients with chronic liver diseases, including MASLD to evaluate liver fibrosis and steatosis. VCTE measures the velocity of shear waves passing through the liver, providing a rapid and accurate method for diagnosing advanced fibrosis. Additionally, it estimates hepatic steatosis through the controlled attenuation parameter (CAP), making it a valuable dual-purpose tool in MASLD management (38).

The AASLD and the EASL recommend VCTE as a first-line imaging modality for fibrosis assessment in MASLD (11, 13, 39). VCTE has demonstrated high sensitivity and specificity for identifying advanced fibrosis (F3-F4) and cirrhosis, particularly in outpatient settings where liver biopsy is not routinely performed. Studies have shown that VCTE has similar prognostic accuracy to liver biopsy for predicting liver-related events (LREs), such as cirrhosis complications and hepatocellular carcinoma (18, 39, 40).

However, VCTE has limitations, particularly in patients with obesity or ascites, where the transmission of ultrasound waves may be compromised (41). In such cases, alternative imaging modalities like magnetic resonance elastography (MRE) may be more appropriate for obtaining accurate measurements. Additionally, VCTE’s accuracy can be affected by factors such as operator skill, acute hepatitis, alcohol abuse, and extrahepatic cholestasis, which may lead to overestimation of liver stiffness. Despite these limitations, VCTE remains a cornerstone in the non-invasive assessment of liver fibrosis and steatosis, providing valuable prognostic information and aiding in the management of patients with MASLD (42, 43).

*The FibroScan-AST (FAST) score* is a composite score that combines liver stiffness measurements obtained from VCTE with serum aspartate aminotransferase (AST) levels to assess the likelihood of advanced fibrosis and MASH. This score is particularly useful for identifying patients with MASH who are at risk of progression to cirrhosis and may be candidates for more aggressive interventions. The FAST score was developed and validated in multiple studies, demonstrating high diagnostic accuracy. A systematic review and meta-analysis by Ravaioli et al., reported that the FAST score has a pooled sensitivity of 89% and specificity of 89% for identifying fibrotic MASH, with a NPV of 92% and a positive predictive value (PPV) of 65% (44, 45). When compared to FIB-3, NFS, APRI, AST score had the highest AUROC for predicting high-risk MASH criteria compared to other non-invasive surrogates, with an AUROC of 0.807 for severe disease with activity ≥3 and/or fibrosis ≥3 (46).

### Magnetic resonance elastography (MRE)

Magnetic resonance elastography (MRE) is considered the gold standard for non-invasive imaging of liver fibrosis. MRE combines traditional magnetic resonance imaging (MRI) with elastography to provide detailed measurements of tissue stiffness, offering superior diagnostic accuracy across all stages of MASLD (47). MRE is especially useful in detecting early fibrosis (F0-F2), where other modalities like VCTE may not be as sensitive (48, 49).

Numerous studies have shown that MRE outperforms VCTE in detecting fibrosis in patients with obesity, a common comorbidity in MASLD, and in distinguishing early-stage fibrosis from more advanced stages (50, 51). MRE performs well in assessing liver stiffness even in the presence of significant liver inflammation. Studies have shown that MRE can effectively distinguish between fibrosis and inflammation, which is particularly useful in the early stages of liver disease where other modalities like vibration-controlled transient elastography (VCTE) may not be as sensitive (52, 53).

Despite its diagnostic superiority, MRE is more expensive and less widely available than VCTE, limiting its routine use to specialized centers. The AASLD and EASL recommend MRE as a second-line imaging option for cases where other non-invasive tests provide inconclusive or unreliable results (11, 13).

### Shear-wave elastography (SWE)

Shear-wave elastography (SWE) is another ultrasound-based modality that measures liver stiffness in real time by evaluating the speed of shear waves generated by ultrasound pulses.

Recent studies have demonstrated the utility of SWE in patients with MASLD, particularly in those with obesity, where traditional methods like VCTE may fail or provide unreliable results, particularly with the use of different transducers (54–56). This becomes particularly relevant and useful given the prevalence of obesity among patients with MASLD.

Severe hepatic steatosis can compromise the accuracy of shear-wave elastography (SWE) by potentially overestimating the degree of liver fibrosis. Additionally, SWE is less reliable in detecting early stages of fibrosis (F0-F1) compared to its higher accuracy in identifying advanced stages (≥F2) (54, 55, 57, 58).

## Discussion

### Comparative performance of non-invasive tests

The comparative performance and recommended use of NITs for assessing liver fibrosis in MASLD vary based on their diagnostic accuracy, ease of use, and clinical context. The FIB-4 Index and the NFS are widely used initial tools due to their high NPV for excluding advanced fibrosis in low-risk patients. However, their sensitivity for detecting early-stage fibrosis is limited, which may result in indeterminate findings that necessitate further imaging-based assessments (59, 60).

The Enhanced Liver Fibrosis (ELF) test consistently outperforms FIB-4 and NFS in terms of sensitivity and specificity for predicting advanced fibrosis and liver-related outcomes, making it a preferred option in tertiary care settings. The ELF test has shown an AUROC of 0.90 for detecting advanced fibrosis, with 80% sensitivity and 90% specificity (61). However, its higher cost and limited availability restrict its widespread use in primary care settings (15). Vibration-controlled transient elastography (VCTE) remains the most widely used imaging modality for non-invasive liver assessment due to its ease of use, immediate results, and ability to assess both liver stiffness and steatosis simultaneously. VCTE has demonstrated high diagnostic accuracy, with an AUROC of 0.85 for advanced fibrosis (60).

Magnetic resonance elastography (MRE) offers superior diagnostic precision, particularly in patients with obesity or early-stage fibrosis, with an AUROC of 0.89 for detecting significant fibrosis (51). However, its high cost and limited accessibility confine its use to specialized centers. Shear wave elastography (SWE) provides an alternative option with comparable accuracy to VCTE but has not yet been widely adopted due to similar cost and availability challenges (62).

The different tests, their intended use, performance and limitations are summarized in Table 1.

**TABLE 1:** Overview of key non-invasive tests (NITs) and imaging modalities for diagnosing MASLD.

Test/Imaging modality	Recommended use	Performance	Limitations
Fibrosis-4 (FIB-4) Score	Initial screening for advanced fibrosis	High negative predictive value (NPV) for excluding advanced fibrosis; recommended for patients with metabolic risk factors.	Limited sensitivity for early-stage fibrosis; intermediate scores require further testing.
NAFLD Fibrosis Score (NFS)	Risk stratification for advanced fibrosis	Cost-effective, easy to calculate, high NPV for ruling out advanced fibrosis.	Limited accuracy in detecting early-stage fibrosis; further assessment often needed.
Enhanced Liver Fibrosis (ELF) Test	Staging of fibrosis, especially advanced stages	High sensitivity (80%) and specificity (90%) for advanced fibrosis.	Higher cost and limited availability, reduced specificity in low-prevalence settings, risk of false positives.
FibroSure (FibroTest)	Assessment of fibrosis and necro-inflammatory activity	High diagnostic accuracy for cirrhosis (AUC 0.92), moderate for advanced fibrosis (AUC 0.77).	Moderate sensitivity and specificity; influenced by acute inflammation and other factors; recommended for use with other modalities.
Vibration-Controlled Transient Elastography (VCTE)	Assessment of liver stiffness and steatosis	High sensitivity and specificity for advanced fibrosis (AUROC 0.85); can assess steatosis using CAP.	Limited accuracy in patients with obesity or ascites; accuracy affected by operator skill and other external factors.
Magnetic Resonance Elastography (MRE)	Gold standard for non-invasive liver fibrosis imaging	Superior accuracy across all fibrosis stages (AUROC 0.89), particularly effective in early-stage fibrosis and obese patients.	Higher cost and less accessibility; typically confined to specialized centers.
Shear-Wave Elastography (SWE)	Real-time liver stiffness assessment	Comparable accuracy to VCTE, especially effective in patients with obesity.	Lower sensitivity for early-stage fibrosis; not as widely adopted due to cost and availability challenges.
FAST Score (FibroScan-AST)	Identification of fibrotic MASH	High sensitivity (89%) and specificity (89%) for fibrotic MASH.	Moderate positive predictive value (PPV); influenced by factors affecting liver stiffness measurement.

### Clinical implications of non-invasive testing

Progression rates in the MASLD spectrum vary significantly, with the fibrosis stage being a critical determinant of clinical outcomes. Patients with advanced fibrosis (F3-F4) are at higher risk for liver-related complications, including cirrhosis, hepatocellular carcinoma, and liver-related mortality. A study by Kaewdech et al., highlighted that all-cause and liver-related mortalities significantly increase from fibrosis stage 2 (*F* ≥ 2) onward, underscoring the importance of early detection and monitoring (63). NIT are therefore recommended by numerous societies and communities for initial risk stratification and longitudinal monitoring of patients with MASLD (11–13, 39, 61).

Non-invasive tests offer several advantages beyond being less invasive than liver biopsy. They facilitate early identification of patients at risk for disease progression, enabling timely intervention and monitoring of treatment response. For instance, the FAST score, which has shown high diagnostic accuracy for identifying fibrotic MASH can allow for more personalized and adaptive treatment strategies (16). Additionally, NITs can guide resource allocation by identifying patients who need subspecialty referrals and optimizing healthcare utilization. Sequential use of NITs, such as combining FIB-4 with VCTE, enhances diagnostic accuracy and reduces unnecessary procedures, making them valuable tools in both primary and specialized care settings (14, 64).

It is worth mentioning that these tests do not inherently differentiate between MASLD and other liver conditions such as alcoholic liver disease (ALD) or hepatitis-induced liver disease. Although effective in predicting fibrosis, these tools lack specificity in distinguishing MASLD from other etiologies, limiting their ability to identify the underlying cause of liver disease (27, 65, 66).

Non-invasive tests also serve as valuable prognostic tools, allowing for better risk assessment and improved patient management. They can predict liver-related events and overall mortality, as demonstrated by Zoncapè et al., who reviewed the use of NITs in the diagnosis and staging of MASLD (14). The use of NITs like the ELF test and VCTE has been shown to correlate with histologic improvements in clinical trials, providing a non-invasive means to monitor treatment efficacy and disease progression (67). This is particularly important with the advent of FDA-approved treatments for MASH, as NITs can help identify patients who will most benefit from these therapies and monitor their response over time (16, 68).

### Future directions

Enhancing the use of NITs in managing MASLD requires a multifaceted approach. First, combining different NITs, as recommended by society guidelines, can improve diagnostic accuracy, specifically through combining serum scores/biomarkers with imaging-based tests (12, 13, 15). Clear clinical pathways will help provide structured assessment, guiding the appropriate use of NITs and optimizing patient care. Second, ongoing research is essential for refining the use of these tests, and developing other tests which may have an added benefit of more specificity to MASLD among other liver disease etiologies. Finally, improving education and awareness among healthcare providers about the utility and interpretation of NITs is crucial. Training programs can help bridge knowledge gaps, equipping both primary care physicians and specialists with the necessary skills to follow the latest guidelines and best practices.

In summary, NITs are indispensable in the management of MASLD, offering high diagnostic accuracy, facilitating early intervention, and enabling dynamic monitoring of disease progression and treatment response. They help optimize healthcare resources by identifying patients who need further evaluation and subspecialty care, ultimately improving patient outcomes and reducing the burden of liver-related complications.
